# Impact of enzalutamide on patient-reported fatigue in patients with prostate cancer: data from the pivotal clinical trials

**DOI:** 10.1038/s41391-021-00447-9

**Published:** 2021-09-13

**Authors:** Bertrand F. Tombal, Stephen J. Freedland, Andrew J. Armstrong, Tomasz M. Beer, Arnulf Stenzl, Cora N. Sternberg, Maha Hussain, Arijit Ganguli, Krishnan Ramaswamy, Hemant Bhadauria, Cristina Ivanescu, James Turnbull, Stefan Holmstrom, Fred Saad

**Affiliations:** 1 Urology, Cliniques universitaires Saint-Luc, UCLouvain, Brussels, Belgium; 2 Urology, Center for Integrated Research in Cancer and Lifestyle, Cedars-Sinai Medical Center, Los Angeles, CA USA; 3 Durham VA Medical Center, Durham, NC USA; 4 School of Medicine, Duke Cancer Institute Center for Prostate & Urologic Cancers, Durham, NC USA; 5 Hematology/Medical Oncology, OHSU Knight Cancer Institute, Oregon Health & Science University, Portland, OR USA; 6 Urology, University Hospital, Eberhard Karls University of Tübingen, Tübingen, Germany; 7 Englander Institute for Precision Medicine, Medical Oncology, Weill Cornell Medicine, Meyer Cancer Center, New York, NY USA; 8 Medicine, Lurie Cancer Center, Northwestern University, Chicago, IL USA; 9 HEOR, Astellas Pharma Inc, Northbrook, IL USA; 10 HEOR, Pfizer Inc, New York, NY USA; 11 Medical Affairs, Astellas Pharma Inc., Northbrook, IL USA; 12 Statistics, IQVIA, Amsterdam-Zuidoost, the Netherlands; 13 Patient Centered Endpoints, IQVIA, New York, NY USA; 14 HEOR, Astellas Pharma Inc., Leiden, the Netherlands; 15 Urology, University of Montréal Hospital Center, Montréal, QC Canada

**Keywords:** Cancer therapy, Prostate cancer

## Abstract

**Background:**

Fatigue is a multifactorial symptom commonly reported by patients with prostate cancer as a result of disease and treatment. This study assesses the impact enzalutamide has on patient-reported fatigue (“fatigue”) by using patient-reported outcomes from four pivotal, placebo-controlled trials of enzalutamide (ARCHES (NCT02677896), PROSPER (NCT02003924), PREVAIL (NCT01212991), and AFFIRM (NCT00974311)).

**Methods:**

Fatigue was assessed in the individual studies using the Functional Assessment of Cancer Therapy–Prostate item GP1 at baseline, weeks 13 or 17, and every 12 weeks until disease progression. Longitudinal changes were assessed using mean scores and mixed-model repeated measures.

**Results:**

The fatigue rates at baseline were higher in patients with later-stage disease (metastatic and/or castration-resistant prostate cancer (CRPC)) and among patients who had already received prior treatment lines; rates ranged between 58% in PROSPER (nonmetastatic CRPC) and 86% in AFFIRM (post-docetaxel metastatic CRPC). Irrespective of disease state, initiation of enzalutamide or placebo resulted in an early increase of fatigue (by weeks 13 or 17), with fatigue levels stabilizing thereafter. At last assessment, ≥55% of patients reported fatigue improvement or stabilization in all trials compared to baseline. More patients reported fatigue worsening by ≥1 or ≥2 units with enzalutamide plus androgen deprivation therapy (ADT) than with placebo plus ADT in ARCHES, PROSPER, and PREVAIL, but the between-group difference was <10% in all trials.

**Conclusions:**

The levels of fatigue were greater in mCRPC and lower in earlier states of disease. In all trials, patients reported a small increase in fatigue for the first 13–17 weeks after starting enzalutamide or placebo, with slightly greater fatigue with enzalutamide in all studies except AFFIRM, but fatigue stabilized or improved thereafter. This suggests a role for clinical management of fatigue to help patients cope early in treatment.

## Introduction

Patients’ experiences with prostate cancer differ throughout the disease continuum, with symptoms worsening as the disease progresses toward metastatic (m) castration-resistant prostate cancer (CRPC) (mCRPC) [1–3]. One of the main symptoms reported by patients with prostate cancer is fatigue [1–3], which can be caused by the disease itself, its treatment, and the advanced age of these patients.

Patients with prostate cancer can already report fatigue at early stages when the disease is localized and they first initiate androgen deprivation therapy (ADT) [4–6]. Patient-reported fatigue may worsen when patients receive chemotherapy or a new hormonal agent in combination with ADT. Fatigue is a common drug-related adverse event (AE) of many prostate cancer drugs such as abiraterone [7], enzalutamide [8], apalutamide [9], docetaxel [10], darolutamide [11], radium-223 [12], cabazitaxel [13], and olaparib [14].

Inconsistencies have been observed between patient- and clinician-reported symptoms, particularly for those symptoms with an important subjective component such as fatigue and pain [15, 16]. A recent report found that rates of patient-reported symptoms such as fatigue differ widely across trials with a similar population with advanced prostate cancer, even among patients treated with placebo, illustrating that differences in data collection methods, patient selection, and geographic regions may influence these outcomes [17]. Fatigue is a multidimensional concept involving psychological and physiological dimensions that can be experienced differently from patient to patient. This subjective experience requires patients to self-report, identify, and describe it [18]. Systematic self-reporting of fatigue by patients over multiple weeks and months of therapy may offer unique insights supplementing clinician-reported fatigue AE.

Enzalutamide is approved in metastatic hormone-sensitive prostate cancer (mHSPC) and CRPC [8]. Within the CRPC setting, enzalutamide has demonstrated efficacy in randomized phase 3 trials in patients with nonmetastatic CRPC (nmCRPC) and mCRPC, both before and after chemotherapy [8]. We used findings of the enzalutamide pivotal phase 3 trials (i.e., ARCHES (NCT02677896) [19], PROSPER (NCT02003924) [20], PREVAIL (NCT01212991) [21], and AFFIRM (NCT00974311) [22]) in these four disease states to assess how rates of baseline patient-reported fatigue vary throughout the disease continuum and the impact of enzalutamide plus ADT vs. placebo plus ADT on patient-reported fatigue using item GP1 (“I have a lack of energy”) of the Functional Assessment of Cancer Therapy–Prostate questionnaire (FACT-P) [23].

## Methods

### Study design

We report the findings of a *post hoc* exploratory analysis of the patient-reported fatigue data from four pivotal, phase 3, randomized, placebo-controlled trials comparing the efficacy and safety of enzalutamide 160 mg per day plus ADT vs. placebo plus ADT. Patient populations differed across studies and included adult men with mHSPC (*n* = 1150) in ARCHES [19], with high-risk nmCRPC (*n* = 1410) in PROSPER, where high risk was defined as prostate-specific antigen doubling time ≤10 months [24], with mCRPC who were asymptomatic or mildly symptomatic after failure of ADT and for whom chemotherapy was not yet clinically indicated (*n* = 1717) in PREVAIL [21], and with mCRPC whose disease has progressed on or after docetaxel therapy (*n* = 1199) in AFFIRM [22]. All four studies were approved by the local ethics committees and conducted in accordance with the principles of the Declaration of Helsinki. All patients gave their signed informed consent before blood collection and data analysis in each trial before random assignment. Further details of the study design and study population included in these studies have been reported in their respective primary efficacy manuscripts [19, 21, 22, 24].

Researchers may request access to anonymized participant-level data, trial-level data, and protocols from Astellas-sponsored clinical trials at www.clinicalstudydatarequest.com. For the Astellas criteria on data sharing, see https://clinicalstudydatarequest.com/Study-Sponsors/Study-Sponsors-Astellas.aspx.

### Patient-reported outcomes (PROs) in the enzalutamide studies

The FACT-P, Brief Pain Inventory–Short Form questionnaire, and EuroQol 5-Dimensions questionnaire were administered in all four studies. In addition, the Brief Fatigue Inventory–Short Form (BFI-SF) was administered in PREVAIL and AFFIRM at baseline, and the prostate cancer module of the European Organisation for Research and Treatment of Cancer Quality of Life questionnaire was administered in ARCHES and PROSPER throughout the study (Supplementary Table 1). The frequency of administration differed across studies (Supplementary Table 1).

Patient-reported fatigue was assessed using the FACT-P item GP1 (“I have a lack of energy”) in all studies. The FACT-P (version 4) reports 27 cancer-specific items in four domains (physical well-being, social or family well-being, emotional well-being, and functional well-being) and 12 prostate cancer-specific items in the prostate cancer subscale to assess function during the previous 7 days [23]. The physical well-being domain has seven items, one of which, GP1, has been mapped to the fatigue concept and has previously been shown to demonstrate strong correlation with the BFI-SF [25]. GP1 is evaluated as a single-item raw score, with a score ranging from 0 (not at all) to 4 (very much).

### Statistical analysis

Analyses were done in the intention-to-treat population (i.e., all patients randomly assigned to study treatment) and based on observed data; no assumptions were made for missing data. The FACT-P completion rate (adjusted for study attrition) at each visit was reported for patients expected to have PRO assessments. Descriptive statistics for the PRO scores, change from baseline, and distribution of change in response categories from baseline were estimated by treatment group and each analysis visit. Statistical comparisons were made using two-sided tests at the *α* = 0.05 significance level, unless stated otherwise. In the distribution analysis, the response categories considered in the analysis were improvement by 1 unit, improvement by 2 units, improvement by ≥3 units, no change/stable, worsening by 1 unit, worsening by 2 units, and worsening by ≥3 units. While the minimal clinically important differences (MCID) have been identified for FACT-P domains, there is no MCID established for item GP1 in the literature. The authors did not attempt to estimate it in the enzalutamide trials.

Longitudinal changes from baseline and from week 13 (or from week 17 in PROSPER) in GP1 scores were analyzed using a restricted maximum likelihood-based mixed-model repeated-measures (MMRM) approach that adjusted for several baseline characteristics (Supplementary Table 2). For the analysis, assessments up to the last visit at which less than 10% of patients in each treatment group had nonmissing data were included to avoid convergence issues. Further details are provided in Supplementary Table 2.

The association of patient-reported fatigue with fatigue AE was assessed for patients on enzalutamide who reported at least one “fatigue” or “asthenia” AE. For each of these patients, the mean score of GP1 during the first fatigue episode, across all fatigue episodes, and during the period with no fatigue was calculated separately. For each patient, all PRO assessments taking place between the start date and end date of a reported fatigue AE were identified. In case of multiple fatigue AE episodes, an average across all episodes was derived for each patient. The mean PRO score during the fatigue period was compared with the mean score during the nonfatigue period using a paired *t*-test. The analysis was conducted separately for fatigue AEs of grade 1, 2, and 3 or higher (3+), according to the Common Terminology Criteria for Adverse Events (CTCAE).

All analyses were conducted using PROC MIXED (SAS^®^ Institute, Cary, NC, USA).

## Results

### Baseline characteristics

Demographics and baseline characteristics were well balanced between arms in all trials, but consistent with the differences in selection criteria, marked differences were observed across studies in baseline disease localization, treatment history, and Eastern Cooperative Oncology Group performance status (Table 1). In addition, baseline FACT-P total score was higher (i.e., better quality of life) for patients in PROSPER and PREVAIL than for patients in ARCHES and AFFIRM (Table 1).

**Table 1:** Demographics and baseline characteristics across pivotal clinical trials.

	ARCHES		PROSPER		PREVAIL		AFFIRM	
	ENZA	PBO	ENZA	PBO	ENZA	PBO	ENZA	PBO
*N*	574	576	933	468	872	845	800	399
*Median age, years (range)*	70 (46–92)	70 (42–92)	74 (50–95)	73 (53–92)	72 (43–93)	71 (42–93)	69 (41–92)	69 (49–89)
*ECOG, %*								
0	78.0	76.9	80	82	67.0	69.2	37	39
1	21.8	23.1	20	18	33	30.8	54	53
≥2	0	0	0	0	0	0	9	8
*Time since diagnosis*								
Mean, months (SD)	17.6 (37.5)	19.99 (41.40)	99.1 (57.3)	94.1 (56.7)	78.6 (59.1)	76.2 (55.7)	86.1 (54.8)	81.9 (50.9)
Median, months (range)	3.5 (0.3–267.9)	3.4 (0.4–259.1)	90.4 (2.2–381.8)	86.8 (2.2–275.7)	62.7 (0.2–326.6)	64.6 (0.1–275.4)	70.9 (5.3–284.6)	71.6 (10.6–268.0)
*Gleason score at diagnosis*								
≤7, %	–	–	54.9	51.7	49.4	47.6	50	48
<8, %	29.8	32.5	–	–				
≥8, %	67.2	64.8	40.8	44.2	50.6	52.4	50	52
Unknown, %	–	–	4.3	4.1	–	–		
Missing, *n*	–	–	–	–	–	–	74	31
*Previous prostatectomy, %*	12.5	15.5	25.08	29.70	25.9	26.6	34.6	30.6
*Previous primary radiation therapy, %*	16.4^a^	16.7^a^	32.58	33.76	39.0	39.1	37.5	41.9
*Previous use of ADT, n (%)*								
None	39 (6.8)	61 (10.6)	34 (3.6)	24 (5.1)	7 (0.8)	7 (0.8)	0	0
1	–	–	320 (34.3)	142 (30.3)	89 (10.2)	94 (11.1)	65 (8.2)	35 (8.8)
2	–	–	339 (36.3)	151 (32.3)	373 (42.8)	360 (42.6)	336 (42.3)	151 (37.9)
3	–	–	164 (17.6)	101 (21.6)	239 (27.4)	237 (28.0)	246 (31.0)	120 (30.2)
≥4	–	–	76 (8.1)	50 (10.7)	164 (18.8)	147 (17.4)	147 (18.5)	92 (23.1)
≤3 months	414 (72.1)	394 (68.4)	–	–	–	–	–	–
>3 months	121 (21.1)	120 (20.8)	–	–	–	–	–	–
*Number of prior DOC regimens, %*								
1	18	18	–	–	–	–	72	74
≥2	–	–	–	–	–	–	28	26
*Geographic scope, %*								
Europe	59	60	49	50	53	53	58	56
North America	15	13	15	13	25	25	33	33
RoW	26	27	36	37	22	23	9	11
*Disease localization at screening, %*								
Bone only	47	43	1	1	40	40	28	31
Soft tissue only	9	8	0	<1	14	18	8	9
Bone and soft tissue	38	42	<1	0	45	42	63	60
None	–	–	99	98	1	3	–	–
*FACT-P, total*								
Mean (SD)	113.9 (19.8)	112.7 (19.0)	119.5 (17.8)	120.8 (16.7)	119.6 (17.8)	119.4 (17.9)	108.7 (21.2)	110.6 (20.8)

*ADT* androgen deprivation therapy, *DOC* docetaxel, *ECOG* Eastern Cooperative Oncology Group, *ENZA* enzalutamide, *FACT-P* Functional Assessment of Cancer Therapy–Prostate, *PBO* placebo, *RoW* rest of world, *SD* standard deviation.

^a^Other included unknown or missing.

### Patient-reported fatigue at baseline

**Fig. 1:**
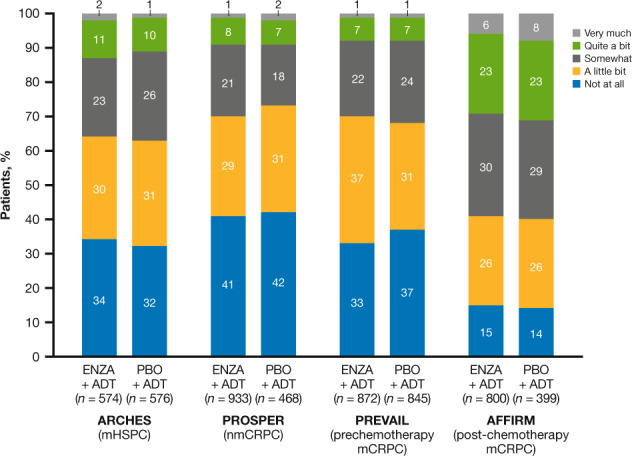
Distribution of baseline GP1 scores across the enzalutamide clinical trials. Item GP1 is a 5-point scale with the following responses: “not at all” (score of 0); “a little bit” (score of 1); “somewhat” (score of 2); “quite a lot” (score of 3); and “very much” (score of 4). The mean (median) score for item GP1 at baseline in the enzalutamide and placebo arms, respectively, was 1.18 (1.00) and 1.18 (1.00) in ARCHES, 1.02 (1.00) and 0.95 (1.00) in PROSPER, 1.06 (1.00) and 1.05 (1.00) in PREVAIL, and 1.79 (2.99) and 1.86 (2.00) in AFFIRM. ENZA enzalutamide, mCRPC metastatic castration-resistant prostate cancer, mHSPC metastatic hormone-sensitive prostate cancer, nmCRPC nonmetastatic castration-resistant prostate cancer, PBO placebo.

Completion rates for FACT-P were high (67–99%) across trials and arms (Supplementary Table 3) [26–29]. Rates of fatigue at baseline (defined as GP1 response of “a little bit” or more, i.e., a score of ≥1) ranged between 58% in patients with nmCRPC (PROSPER) and 85% in patients with post-chemotherapy mCRPC (AFFIRM). Baseline scores for FACT-P item GP1 across studies indicate mild fatigue at study entry, with a median score of 1.0, except for the AFFIRM population, for whom the baseline median score was 2.0. The proportion of patients with severe fatigue at baseline, defined as a GP1 score of 3 or greater, was highest in AFFIRM (29–31% vs. 8–13% in the other three trials, Fig. 1).

### Change from baseline in fatigue

**Fig. 2:**
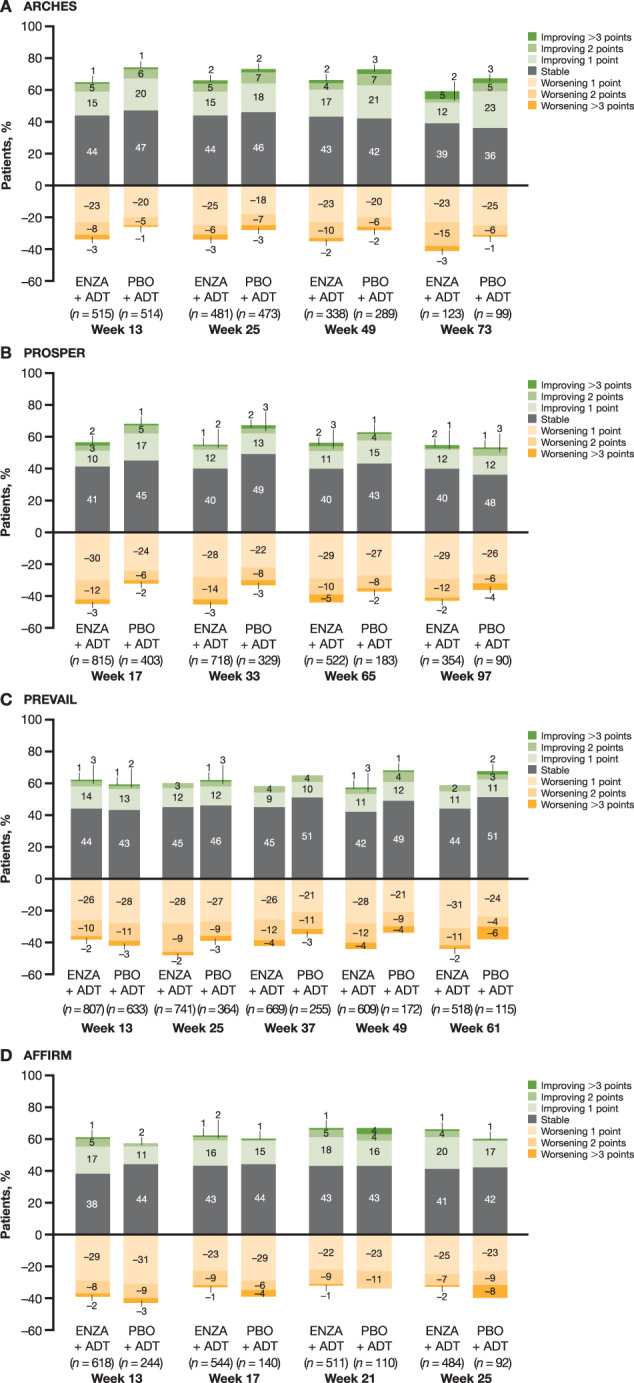
Distribution of change of responses from baseline for item GP1, “I have a lack of energy.” **A** ARCHES, **B** PROSPER, **C** PREVAIL, **D** AFFIRM. ENZA enzalutamide, PBO placebo.

The results of the distribution of change from baseline for FACT-P item GP1 showed that >50% of patients experienced a stable level of fatigue throughout the study or reported improvement by ≥1 GP1 unit in both treatment arms across all four trials (Fig. 2). In ARCHES, PROSPER, and PREVAIL, all of which included fewer symptomatic patients than AFFIRM, more patients experienced worsening of fatigue by ≥1 GP1 unit with enzalutamide plus ADT than with placebo plus ADT across the study. In contrast, in AFFIRM, which included more symptomatic patients, fewer patients experienced fatigue worsening by ≥1 unit with enzalutamide plus ADT compared with placebo plus ADT (Fig. 2). The percentage of patients reporting fatigue worsening by ≥1 GP1 unit was 23–25% and 18–25% across time assessments with enzalutamide plus ADT and placebo plus ADT, respectively, in ARCHES, 28–30% and 22–27%, in PROSPER, 26–31% and 21–28%, in PREVAIL, and 22–29% and 23–31%, in AFFIRM. The percentage of patients reporting fatigue worsening by ≥3 GP1 units was ≤8% across all time points in both arms and all trials (Fig. 2).

At the last assessment time point, the percentage of patients reporting worsening in fatigue relative to baseline by ≥1 or ≥2 units was higher with enzalutamide plus ADT than with placebo with ADT in ARCHES (between-group difference for ≥1 unit: +9%, between-group difference for ≥2 units: +12%), PROSPER (+9%, +5%), and PREVAIL (+10%, +3%), but lower in AFFIRM (–6%, –8%). More patients reported worsening by ≥3 units with enzalutamide plus ADT than with placebo plus ADT in ARCHES (between-group difference: +2%), while fewer patients reported similar worsening with enzalutamide plus ADT than with placebo plus ADT in PROSPER (–2%), PREVAIL (–4%), and AFFIRM (–6%). However, the difference between arms was small in all trials (Table 2). Most patients who reported worsening in fatigue had only a 1-unit increase in GP1 in both arms in all trials. Regarding improvement relative to baseline, fewer patients in the placebo plus ADT arm than in the enzalutamide plus ADT arm experienced improvement in their fatigue by ≥1 or ≥2 units in GP1 in ARCHES (≥1 unit: –12%, ≥2 units: –1%), PROSPER (–1%, –2%), and PREVAIL (–2%, –2%), but more patients in the placebo plus ADT arm than in the enzalutamide plus ADT arm experienced similar improvement in AFFIRM (+7%, +5%).

**Table 2:** Percentage of patients with worsening or improvement of fatigue by EoS.

Study	EoS (weeks)	Worsening						Improvement					
		≥1 point		≥2 points		≥3 points		≥1 point		≥2 points		≥3 points	
		ENZA	PBO	ENZA	PBO	ENZA	PBO	ENZA	PBO	ENZA	PBO	ENZA	PBO
ARCHES	73	41.5	32.4	18.7*	7.1*	3	1	19.5	31.3	7.3	8.1	5	3
PROSPER	97	44.1	35.6	14.7	10.0	2	4	15.8	16.6	3.4	4.4	2	1
PREVAIL	61	43.0	33.1	12.5	9.6	2	6	13.3	15.6	2.1	4.3	0	2
AFFIRM	25	33.3	39.1	8.5*	16.3*	2	8	25.3	18.5	5.7	1.1	1	1

*ENZA* enzalutamide, *EoS* end of study, *PBO* placebo.

*n* = % unless otherwise specified.

*Between-group *p* < 0.05.

### Adjusted change from baseline and weeks 13 or 17 (MMRM analysis)

**Fig. 3:**
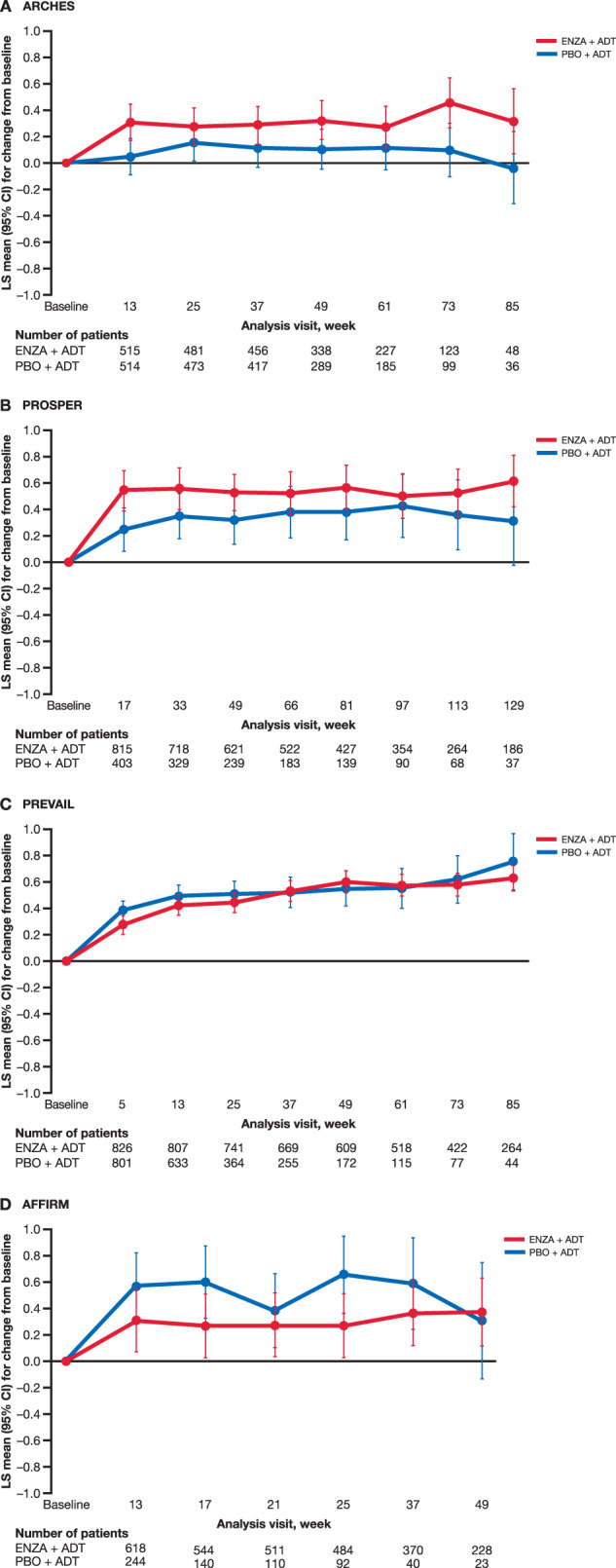
MMRM change from baseline for item GP1. **A** ARCHES, **B** PROSPER, **C** PREVAIL, **D** AFFIRM. CI confidence interval, ENZA enzalutamide, LS least squares, MMRM mixed-model repeated measures, PBO placebo.

The analysis included assessments up to the last visit at which <10% of patients in each treatment group had nonmissing data to avoid convergence issues. An adjusted mean increase from baseline in GP1 score was observed by week 13 (week 17 in PROSPER) in both arms across all studies (Fig. 3). At week 13 (week 17 in PROSPER), the increase in score was greater in the enzalutamide arm in ARCHES (enzalutamide: +0.31, placebo: +0.05, difference: 0.26, 95% confidence interval (CI): 0.14–0.38) and PROSPER (enzalutamide: +0.55, placebo: +0.25, difference: 0.30, 95% CI: 0.18–0.41) but greater in the placebo arm in PREVAIL (enzalutamide: +0.42, placebo: +0.49, difference: –0.07, 95% CI: –0.17 to 0.03) and AFFIRM (enzalutamide: +0.31, placebo: +0.57, difference: –0.2, 95% CI: –0.41 to –0.11). The initial increase was <1 unit in all arms and in all trials.

Change from baseline stabilized after week 13 (week 17 in PROSPER) in both arms across all studies. The overall adjusted mean increase in score from baseline throughout the study was significantly higher for enzalutamide plus ADT than for placebo plus ADT in ARCHES (between-group difference: 0.23, 95% CI: 0.12–0.35) and PROSPER (between-group difference: 0.20, 95% CI: 0.10–0.30), but significantly lower in AFFIRM (between-group difference: –0.21, 95% CI: –0.36 to –0.06). Despite reaching statistical significance, these between-group differences were small. No differences were observed between arms in PREVAIL (difference: –0.04, 95% CI: –0.13 to 0.05, Fig. 3).

A sensitivity analysis was conducted to assess change from week 13 (week 17 in PROSPER, Supplementary Fig. 1). Fatigue did not significantly increase after weeks 13 or 17 in any arm and all changes were <0.4 points. In all trials, any worsening in fatigue from weeks 13 or 17 to end of treatment was similar or significantly lower for enzalutamide plus ADT than for placebo plus ADT.

### Association between patient-reported fatigue and fatigue AE

**Fig. 4:**
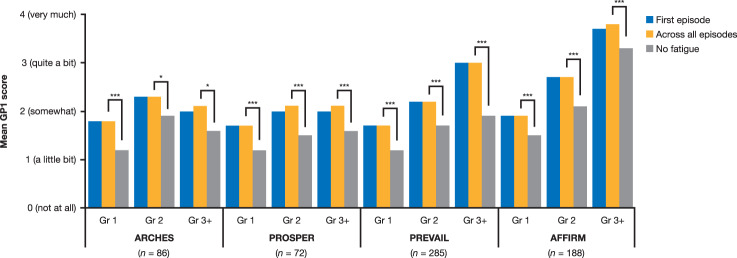
Mean FACT-P GP1 score during the period of a fatigue AE and outside this period. First episode = the period of the first fatigue AE; across all episodes = the period of any fatigue AE; no fatigue = the period when no fatigue AE was reported. AE adverse event, FACT-P Functional Assessment of Cancer Therapy–Prostate, Gr grade. *p < 0.01; ***p < 0.0001.

While patient-reported fatigue provides a more systematic and planned assessment of fatigue over time, we also assessed if the spontaneous reports captured by the clinician (i.e., fatigue AE; Supplementary Table 4) correlated with patient-reported fatigue. In the enzalutamide plus ADT arm, the mean scores for GP1 during fatigue AE were significantly higher than the mean scores for the period with no AE in all four studies, irrespective of the fatigue AE grade (Fig. 4). No differences were observed for the GP1 scores associated with the first vs. all fatigue episodes. However, while the scores for GP1 for grade 2 AE were higher than for grade 1 AE in all studies, GP1 scores for grade 3+ AE were only higher than those for grade 2 AE in PREVAIL and AFFIRM; no differences were observed between grade 2 and grade 3+ AE in ARCHES and PROSPER. The mean GP1 scores during the period of no AE fatigue ranged between 1.2 for patients who had experienced a grade 1 fatigue AE in ARCHES, PROSPER, and PREVAIL and 3.3 for patients who had experienced a grade 3 fatigue AE in AFFIRM, suggesting that some patients may have experienced fatigue beyond the duration of the fatigue AE.

An indicator of the level of toxicity of AEs is the proportion of these events leading to dose reductions or treatment discontinuation. The proportion of patients with a fatigue-related AE leading to dose interruption, dose reduction, or treatment discontinuation was ≤2.8%, ≤3.9%, and ≤1.6% of patients in the enzalutamide arm in the individual studies, respectively. These rates were ≤0.9%, ≤0.6%, and ≤1.3%, respectively, in the placebo arm (Supplementary Table 5). For patients with an asthenia AE, these rates were lower (data not shown).

## Discussion

Baseline fatigue rates in the four pivotal enzalutamide trials suggest that >50% of patients experience fatigue across the disease continuum, with the highest rates reported by patients with mCRPC who have progressed during or after docetaxel. The highest prevalence in AFFIRM may be due to a more metastatic and heavily pretreated castration-resistant disease state and to the previous exposure to docetaxel [22].

Of the four trials, the population with the most recent diagnosis of prostate cancer was in ARCHES (median time since diagnosis: 3.4–3.5 months). However, the rate of fatigue in ARCHES was already quite high at baseline prior to enzalutamide initiation (66–68%) partly due to exposure to ADT and partly to the burden of metastatic disease. Almost all patients (91%) had already initiated ADT prior to study entrance, with 70% of patients having initiated ADT within the previous 3 months. Initiation of ADT has been reported to trigger fatigue in most patients [6]. Baseline rate of fatigue in ARCHES was similar to that in PROSPER and PREVAIL.

The high rates of baseline fatigue in the enzalutamide pivotal trials may also be partly due to the advanced age of these patients (median age: 69–74 years). Prevalence of fatigue in the general population has been reported in up to 29% and 53% of community-living adults aged 70 and 78, respectively [30].

Initiation of enzalutamide was associated with an early increase in fatigue in all studies. The early increase was also observed for patients initiating treatment with placebo, even in PROSPER, PREVAIL, and AFFIRM, where >65% of patients had been exposed to at least two types of ADT for a long time before entering the study. The magnitude of the initial and overall worsening of fatigue was small in all trials, with most patients reporting worsening by one GP1 unit only. By the last assessment, more patients reported fatigue worsening by ≥1 or ≥2 units in GP1 with enzalutamide plus ADT than with placebo plus ADT in ARCHES, PROSPER, and PREVAIL and by ≥3 units in ARCHES. However, all differences between treatment arms were small. At the end of the study, the percentage of patients with worsening of fatigue by one or more GP1 units in the enzalutamide plus ADT and in the placebo plus ADT arms was 42% and 32% (difference: +9%), respectively, in ARCHES, 44% and 36% in PROSPER (difference: +9%), 43% and 33% (difference: +10%) in PREVAIL, and 33% and 39% (difference: –6%) in AFFIRM. For improvement of fatigue (by ≥1 unit), these proportions were 20% and 31% (difference: –12%) in ARCHES, 16% and 17% (difference: –1%) in PROSPER, 13% and 16% (difference: –2%) in PREVAIL, and 25% and 19% (difference: +7%) in ARCHES.

In all studies and treatment arms, the level of fatigue remained mostly stable from weeks 13 and 17 to the end of treatment after the initial increase. The early worsening of fatigue observed with enzalutamide plus ADT was greater than with placebo plus ADT in ARCHES and PROSPER, which included relatively asymptomatic patients. In contrast, the early worsening of fatigue was more pronounced with placebo in PREVAIL and AFFIRM.

In all four pivotal trials, the GP1 score increased significantly when patients experienced a fatigue AE, irrespective of the CTCAE grade, indicating that patient-reported fatigue and fatigue AE are correlated and measuring the same concept of fatigue. However, no consistent association was observed between the CTCAE grade of fatigue and the GP1 score, possibly because of a small number of higher-grade events. In addition, patients who experienced a fatigue AE had mean GP1 scores ≥1.0, even when not during the fatigue episode in all studies, suggesting misalignment between patient-reported fatigue and fatigue AE, i.e., in some cases, there is a misalignment between what the patients report in a systematic assessment and what is captured by the physician on the basis of a spontaneous AE report. Patient-reported fatigue may provide a richer and more nuanced picture of fatigue manifestation, severity, time course, and resolution. Similar inconsistencies have also been highlighted by others [15].

Rates of fatigue AEs leading to dose interruption or reduction, or treatment discontinuation, were low in all arms and in all trials suggesting that, in general, fatigue was not severe or may have been manageable with other strategies. Several strategies are currently used to prevent or manage fatigue. In the case of cancer patients experiencing fatigue, the European Society for Medical Oncology recommends early diagnosis and implementation of one or more of the following strategies: counseling to patient and family, physical exercise of moderate intensity, yoga, psychoeducation, and, if metastatic disease, short-term use of steroids (dexamethasone or methylprednisolone) [31]. In the enzalutamide studies, concomitant use of systemic steroids was reported in both treatment arms in all trials with the highest rates being observed in PREVAIL (enzalutamide: 26.5%; placebo: 30.2%) and AFFIRM (enzalutamide: 47.8%; placebo: 45.6%). However, the markedly higher use of steroids in these trials may also be related to the more advanced disease stage in these patients and the need for steroids to palliate pain or reduce inflammation. Overall, these strategies may help patients who initiate treatment with enzalutamide or other prostate cancer therapies to cope with fatigue early in treatment.

Our study has some limitations. None of the studies followed patients across the full disease continuum; however, the four pivotal trials encompass most states of the disease and provide a comprehensive portrait of the disease evolution. Nevertheless, none of the studies continued to collect PRO or patient-reported fatigue data after disease progression, thus attenuating any changes in fatigue levels that may impact patients after such an event; however, this is a limitation that is mostly biased against enzalutamide, as patients in the ADT arm typically progressed quicker. Another limitation is that the treatment history of patients in the clinical studies conducted earlier (AFFIRM and PREVAIL) may not reflect current treatment pathways. Management of patients with prostate cancer has experienced ongoing intensification in the last few years with earlier use of therapies such as abiraterone, docetaxel, and enzalutamide, which, until recently, had been restricted to the most advanced stages of the disease. Earlier use of these therapies, particularly of docetaxel, may have an impact on the baseline prevalence of patient-reported fatigue in patients with mCRPC.

Another limitation is the use of a single item (FACT-P item GP1) to assess patient-reported fatigue, which is a multidimensional concept. However, FACT-P item GP1 showed a good correlation with the BFI-SF [25] and fatigue AE and, moreover, should not have affected comparisons, as the same instrument was used in both treatment arms across all studies. The lack of established MCID for GP1 may make the interpretation of the findings difficult. However, none of the mean differences between enzalutamide plus ADT and placebo plus ADT were larger than 1, i.e., the minimum change a patient can report in GP1, suggesting that none of the differences observed for any arm were clinically meaningful. Similar to other PRO analyses, a ceiling-floor effect was observed for GP1. However, this effect impacted both arms in all studies equally. In addition, a larger proportion of patients had a score of 0 (floor) than of 4 (ceiling) at baseline, suggesting that the effect would mainly impact improvement rather than worsening of fatigue.

Lastly, another limitation is that our analyses included observed data only; no imputations or adjustments were made for missing data. Patients who may not have completed the FACT-P because of fatigue or other type of AE that may have affected the GP1 score were not measurably accounted in our analysis. However, the high completion rates, particularly in the enzalutamide arm (≥88% across all visits in all trials), mitigate this concern.

Our study also had several strengths. This is a novel analysis to assess longitudinal changes of fatigue across the disease continuum in a randomized, controlled setting across multiple centers. Our analysis is based on a very large sample size (5467 patients). The double-blind nature of the four pivotal studies removes the potential bias of open-label studies.

Our analyses suggest that fatigue is common across the disease continuum, with the highest rates among patients with mCRPC progression on or after docetaxel. Patient-reported fatigue increases within the first 3 or 4 months of initiating treatment with enzalutamide but remains stable thereafter. This suggests a role for clinical management of fatigue in those patients to enable them to cope with treatment intensification in light of the superior clinical outcomes that result by adding enzalutamide to ADT. In patients with the greatest burden of cancer, fatigue increases seem to be greater with placebo than with enzalutamide, suggesting that effective cancer treatment may reduce cancer-related fatigue. Conversely, in studies of asymptomatic patients with lower cancer burden, fatigue increases modestly more with initiation of enzalutamide than with placebo. The relationship between fatigue AE and GP1 scores suggests good correlation, but a discrepancy with patients reporting fatigue beyond the AE episode. Systematic collection of patient-reported fatigue and assessment of how bothersome it is to patients should be considered in clinical trial design in addition to CTCAE AE to have a more comprehensive and nuanced view on the true impact of cancer-related fatigue on patients.

## Supplementary information

Tombal et al. Supplementary

## Data Availability

Researchers may request access to anonymized participant-level data, trial-level data, and protocols from Astellas-sponsored clinical trials at www.clinicalstudydatarequest.com. For the Astellas criteria on data sharing, see https://clinicalstudydatarequest.com/Study-Sponsors/Study-Sponsors-Astellas.aspx.
