# Supplementary material for: Impact of enzalutamide on patient-reported fatigue in patients with prostate cancer: data from the pivotal clinical trials
Source: Prostate Cancer Prostatic Dis. 2021 Sep 13;25(2):288–95. doi: 10.1038/s41391-021-00447-9 (PMC9184276; doi:10.1038/s41391-021-00447-9)
Supplement: Supplementary file 1 — Tombal et al. Supplementary [file 41391_2021_447_MOESM1_ESM.pdf]

Supplementary information

Supplementary Table 1 PRO in enzalutamide studies.

**Study**	**FACT-P**	**QLQ-PR25**	**BPI-SF**	**BFI**	**EQ-5D**	**PRO collection**
ARCHES	X	X	X	–	X (5L)	D1, W13, Q12W
PROSPER	X	X	X	–	X (5L)	D1, W17, Q16W
PREVAIL	X[a]	–	X	X (baseline[b])	X (3L)	D1, W13, Q12W
AFFIRM	X	–	X (D1, D13[b])	–	X (3L; baseline[b])	D1, W13, W17, W21, W25, Q12W

3L 3-level version of the EQ-5D, *5L* 5-level version of the EQ-5D, *D* day, *FACT-P* Functional Assessment of Cancer Therapy–Prostate, *QLQ-PR25* prostate cancer module of the European Organisation for Research and Treatment of Cancer Quality of Life questionnaire, *BPI-SF* Brief Pain Inventory–Short Form, *BFI* Brief Fatigue Inventory, *EQ-5D* EuroQol 5-Dimensions questionnaire, *PRO* patient-reported outcome, *Q12W* every 12 weeks, *Q16W* every 16 weeks, *W* week.

[a]FACT-P was also administered at week 5.

[b]Only administered at these time points.

Supplementary Table 2. Fixed effects included in the longitudinal model.

Study	Randomization factors and additional covariates
ARCHES	Age (continuous)
	Region (North America, Europe, RoW)
	Pain score at baseline based on question 3 of the BPI
	ECOG status at baseline (categorical 0 vs. 1)
	Time from initial diagnosis of prostate cancer to randomization (continuous)
	Volume of disease (low vs. high)
	Prior docetaxel use (yes vs. no)
	Prior use of ADT (none, ≤3 months, >3 months)
	Localization of metastasis based on ICR (bone only, soft tissue only, both bone and soft tissue, none)
	Number of bone lesions (continuous)
	Prior radiation (yes vs. no)
	Previous surgery (yes vs. no)
PROSPER	Age (continuous)
	Region (North America, Europe, RoW)
	Pain score at baseline based on question 3 of the BPI
	ECOG status at baseline (categorical 0 versus 1)
	PSA doubling time (categorical <6 months or ≥6 months)
	Number of prior hormonal therapies (continuous)
	Time (months) from initial diagnosis of prostate cancer to randomization (continuous)
	Prior or current use of a bone targeting agent (yes vs. no)
	Disease status (metastatic vs. nonmetastatic)
	Number of unique prior prostate cancer therapies
	Number of unique prior hormonal therapies
PREVAIL	Age (continuous)
	Region (North America, Europe, RoW)
	Pain score at baseline based on question 3 of the BPI
	Fatigue severity at baseline using the BFI (none vs. mild vs. moderate vs. severe)
	ECOG status at baseline (categorical 0 vs. 1)

Study	Randomization factors and additional covariates
	Number of bone metastases (continuous)
	Number of previous cancer therapies
	Disease localization at screening (bone only, soft tissue only, both bone and soft tissue, none)
AFFIRM	Age (continuous)
	Region (North America, Europe, RoW)
	Pain score at baseline based on question 3 of the BPI
	Fatigue severity at baseline using the BFI (none versus mild vs. moderate vs. severe)
	ECOG status at baseline (categorical 0–1 vs. 2)
	Number of prior chemotherapy regimens (1 vs. ≥2)
	Time (months) from initial diagnosis of prostate cancer to randomization (continuous)
	Number of bone metastases (continuous)
	Disease localization at screening (bone only, soft tissue only, both bone and soft tissue, none)

ADT androgen deprivation therapy, *BFI* Brief Fatigue Inventory, *BPI* Brief Pain Inventory, *CI* confidence interval, *ECOG* Eastern Cooperative Oncology Group, *ICR* independent committee review, *LS* least squares, *PRO* patient-reported outcome, *PSA* prostate-specific antigen, *RoW*, rest of world, *SE* standard error.

The model included the patient as the random effect and several fixed-effects covariates (treatment arm, randomization factors, and additional baseline covariates, number of comorbidities at baseline, use of opioids or corticosteroids during study, and baseline PRO score). The LS mean estimates, SE, 95% CI, and *p* values (where applicable) for mean change from baseline to each visit and across all time points, giving each visit equal weight, were estimated for within-treatment groups and differences between treatments.

Supplementary Table 3. Completion rates for FACT-P across studies.

Study	FACT-P completion rate, *n/N* (%)							
	**Baseline**	**Week 13**	**Week 17**	**Week 25**	**Week 49**	**Week 61**	**Week 73**	**Week 97**
ARCHES								
ENZA	550/572 (96)	533/572 (93)	–	499/535 (93)	340/391 (89)	236/265 (89)	128/146 (88)	–
PBO	553/574 (96)	529/574 (92)	–	487/530 (92)	298/332 (90)	191/213 (90)	101/115 (88)	–
PROSPER								
ENZA	887/933 (95)	–	841/888 (95)	–	637/685 (93)	–	–	365/389 (94)
PBO	439/468 (94)	–	420/444 (95)	–	250/268 (93)	–	–	96/103 (93)
PREVAIL								
ENZA	865/872 (99)	821/835 (98)	–	756/777 (97)	619/643 (96)	528/554 (95)	429/457 (94)	182/192 (95)
PBO	834/845 (99)	643/653 (99)	–	372/387 (96)	177/185 (96)	118/129 (92)	80/87 (92)	27/28 (96)

Study	FACT-P completion rate, *n/N* (%)							
	**Baseline**	**Week 13**	**Week 17**	**Week 25**	**Week 49**	**Week 61**	**Week 73**	**Week 97**
AFFIRM								
ENZA	783/800 (98)	645/672 (96)	–	503/531 (95)	237/269 (88)	120/136 (88)	53/58 (91)	1/1 (100)
PBO	394/399 (99)	254/264 (96)	–	95/103 (92)	23/28 (82)	8/12 (67)	5/6 (83)	–

ENZA enzalutamide, *FACT-P* Functional Assessment of Cancer Therapy–Prostate, *PBO*, placebo.

n = number of patients with evaluable response forms; *N* = total number of patients available to be assessed at a given time point (evaluable questionnaires being those with sufficient data for the calculation of at least one subscale).

Supplementary Table 4. Fatigue AE in the clinical trials.

	ARCHES		PROSPER		PREVAIL		AFFIRM	
	**ENZA**	**PBO**	**ENZA**	**PBO**	**ENZA**	**PBO**	**ENZA**	**PBO**
N	572	574	930	465	871	844	800	399
Fatigue TEAE, *n* (%)								
All grades	112 (19.6)	88 (15.3)	303 (32.6)	64 (13.8)	310 (35.6)	218 (25.8)	269 (33.6)	116 (29.1)
Grade 3+	5 (0.9)	6 (1.0)	27 (2.9)	3 (0.6)	16 (1.8)	16 (1.9)	50 (6.3)	29 (7.3)
Asthenia TEAE, *n* (%)								
All grades	31 (5.4)	28 (4.9)	82 (8.8)	28 (6.0)	113 (13.0)	67 (7.9)	140 (17.5)	67 (16.8)
Grade 3+	6 (1.0)	3 (0.5)	11 (1.2)	1 (0.2)	11 (1.3)	8 (0.9)	20 (2.5)	10 (2.5)

AE adverse event, *ENZA* enzalutamide, *PBO* placebo, *TEAE* treatment-emergent adverse event.

Supplementary Table 5. Fatigue AEs leading to dose interruption, dose reduction, or treatment discontinuation in the clinical trials.

	**ARCHES**		**PROSPER**		**PREVAIL**		**AFFIRM**	
	**ENZA**	**PBO**	**ENZA**	**PBO**	**ENZA**	**PBO**	**ENZA**	**PBO**
N	572	574	930	465	871	844	800	399
Any fatigue-related event leading to dose interruption, n (%)	3 (0.5)	1 (0.2)	26 (2.8)	1 (0.2)	9 (1.0)	8 (0.9)	12 (1.5)	2 (0.5)
Any fatigue-related event leading to dose reduction, n (%)	8 (1.4)	3 (0.5)	36 (3.9)	3 (0.6)	5 (0.6)	1 (0.1)	4 (0.5)	1 (0.3)
Any fatigue-related event as primary reason for treatment discontinuation, n (%)	2 (0.3)	2 (0.3)	15 (1.6)	0	2 (0.2)	11 (1.3)	5 (0.6)	2 (0.5)

AE adverse event, *ENZA* enzalutamide, *PBO* placebo, *TEAE* treatment-emergent adverse event.

A ARCHES

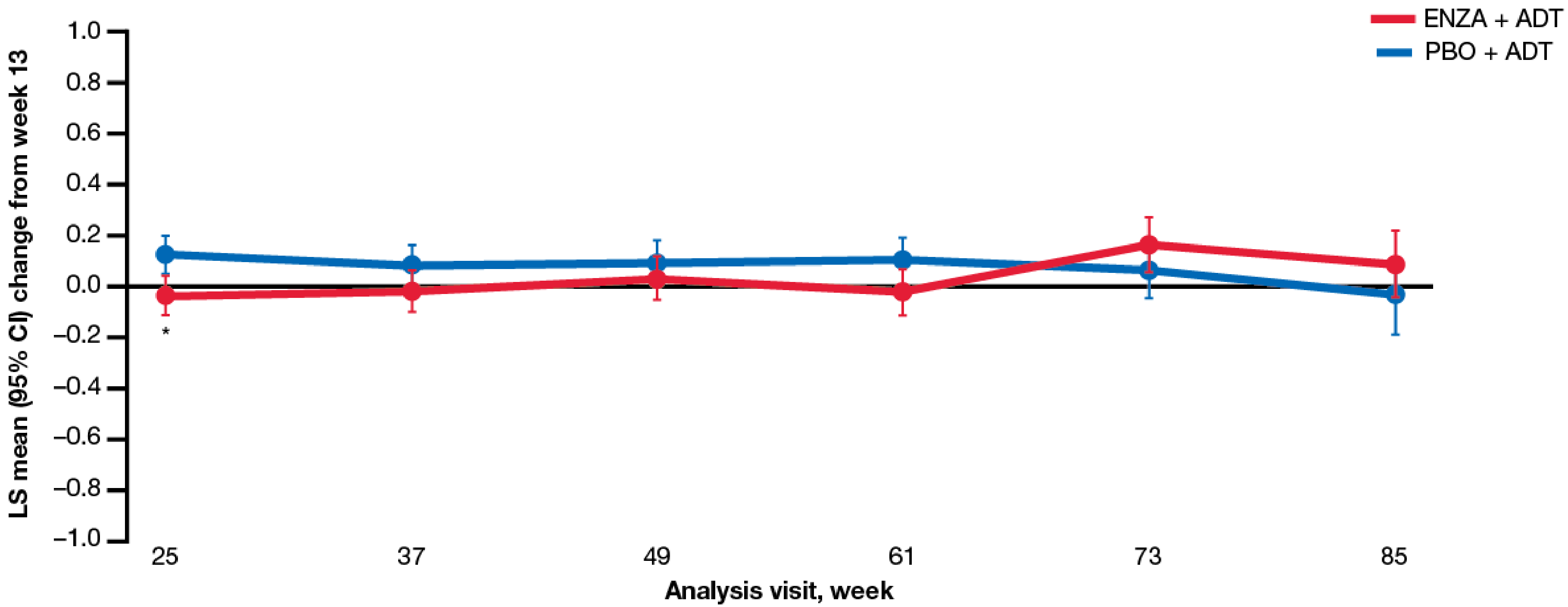

Number of patients						
ENZA + ADT	481	456	338	227	123	48
PBO + ADT	473	417	289	185	99	36

B PROSPER

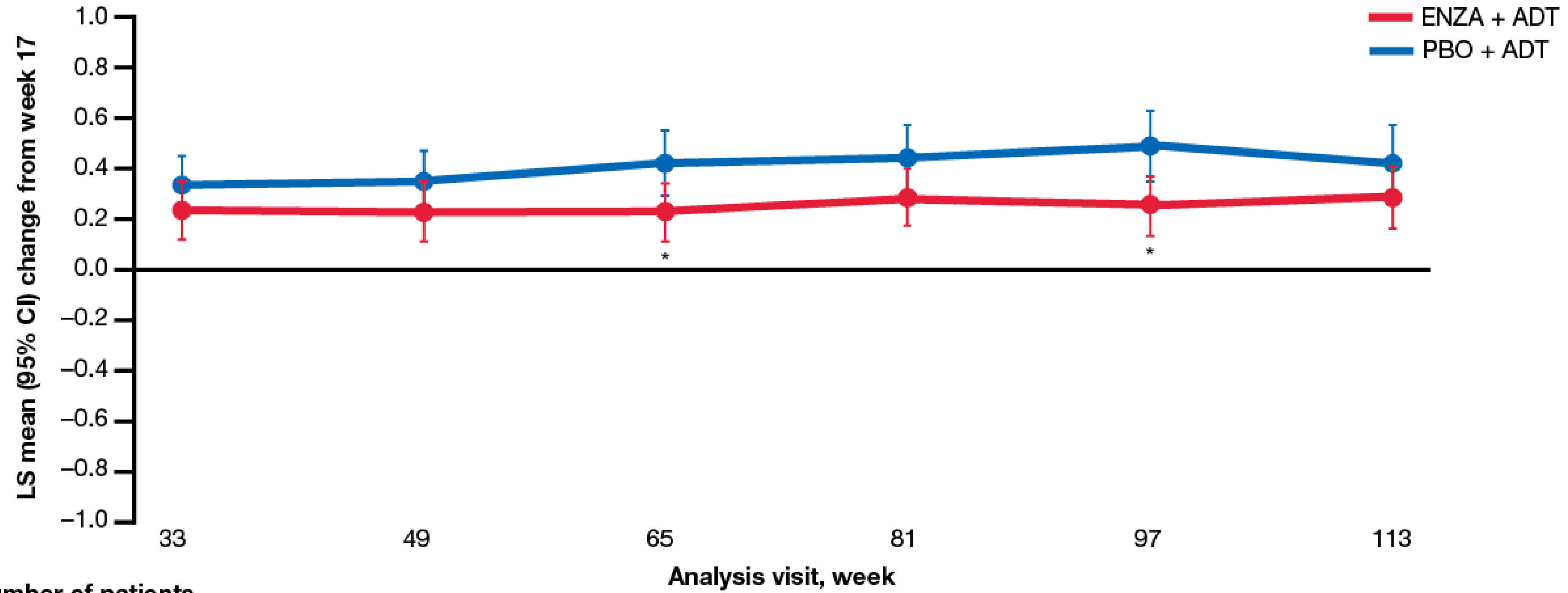

Number of patients						
ENZA + ADT	718	621	522	427	354	264
PBO + ADT	329	239	183	139	90	68

C PREVAIL

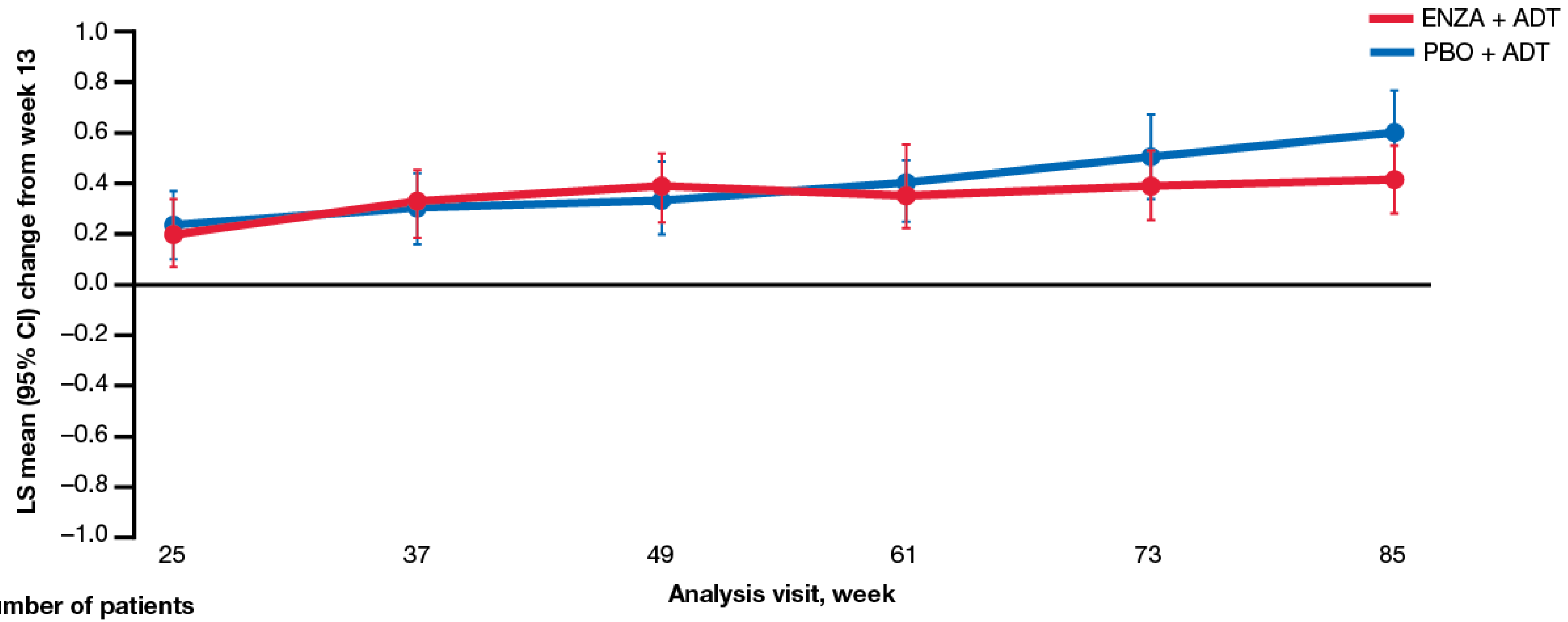

Number of patients

ENZA + ADT	741	669	609	518	422	264
PBO + ADT	364	255	172	115	77	44

D AFFIRM

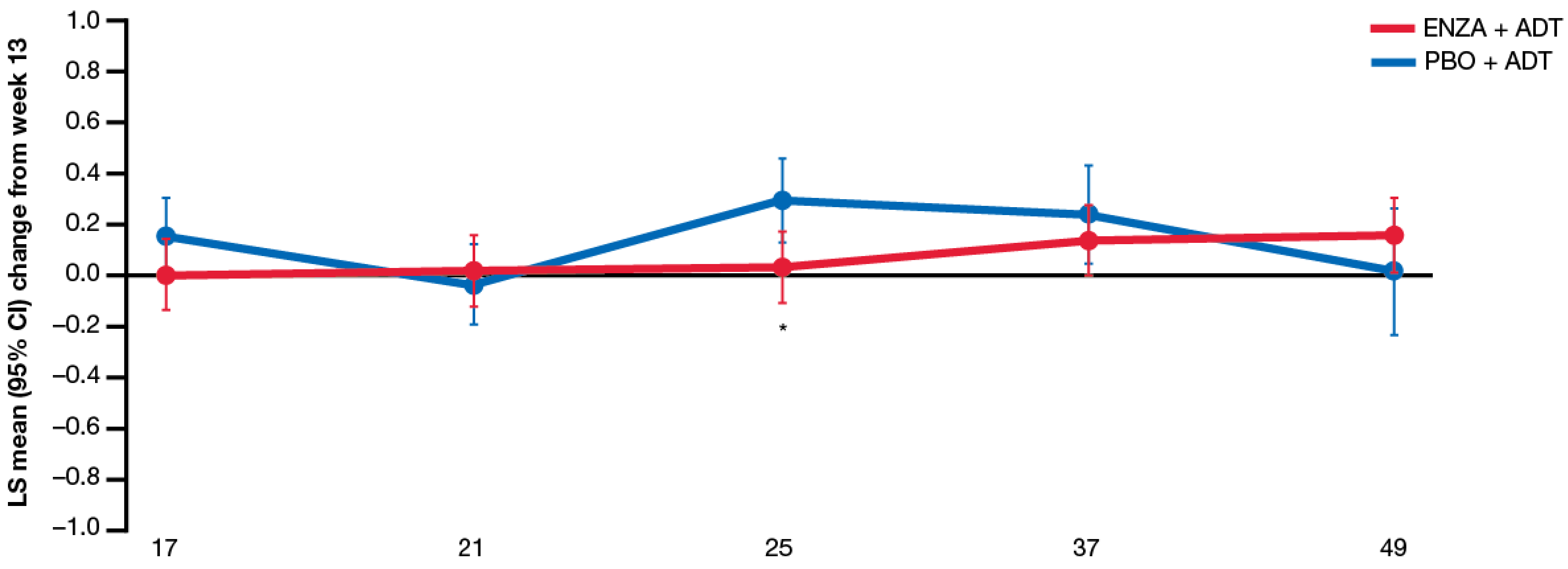

Number of patients					
ENZA + ADT	544	511	484	370	228
PBO + ADT	244	110	92	40	23

Supplementary Fig. 1 MMRM change from week 13 (and week 17 in PROSPER) for item GP1. *CI* confidence interval, *ENZA* enzalutamide, *LS* least squares, *MMRM* mixed-model repeated measures, *PBO* placebo. *$p < 0.05$.
